# Validity of muscular fitness self-assessments in the *ecofit* smartphone application: A correlation study

**DOI:** 10.1371/journal.pone.0278374

**Published:** 2022-12-01

**Authors:** Anna K. Jansson, David R. Lubans, Mitch J. Duncan, Jordan J. Smith, Adrian Bauman, John Attia, Ronald C. Plotnikoff

**Affiliations:** 1 Centre for Active Living and Learning, School of Education, University of Newcastle, Callaghan, New South Wales, Australia; 2 Active Living Research Program, Hunter Medical Research Institute, New Lambton Heights, New South Wales, Australia; 3 School of Medicine and Public Health, University of Newcastle, Callaghan, Australia; 4 School of Public Health, University of Sydney, Camperdown, New South Wales, Australia; Anglia Ruskin University, UNITED KINGDOM

## Abstract

**Background:**

Mobile app-based interventions have the potential for wide-reach and therefore may be a useful tool in up-scaling physical activity interventions. In larger-scale interventions, face-to-face assessments are less cost-effective, and researchers often rely on surveys or activity trackers to assess outcomes. However, there is limited evidence of valid muscular fitness assessments that can be self-administered within mHealth interventions. As such, this study will evaluate the concurrent validity of upper and lower body muscular fitness that have been independently assessed by participants via the *ecofit* app, and face-to-face assessments conducted by a trained researcher.

**Methods:**

This study compared baseline data from two muscular fitness tests from the *ecofit* two-armed randomised controlled trial and self-assessed data collected via the *ecofit* smartphone app (i.e., validated 90-degree push-up and 60-second sit-to-stand test). To assess the concurrent validity, the self-assessed push-up and sit-to-stand tests (i.e., collected via the *ecofit* app) were correlated using Spearman’s correlation coefficient against the researcher-assessed results (i.e., objective results collected during baseline assessment for the *ecofit* trial). Bland-Altman plots were also used to allow visualisation of the differences between the self- and research-assessed tests.

**Results:**

Participants (N = 54) completed the push-up (24.1%) and sit-to-stand (100%) tests within 14-days of receiving the app. The results revealed a strong significant correlation for the push-up test (0.83, *p*<0.001) and a moderate significant correlation for the sit-to-stand test (0.63, *p*<0.001).

**Conclusion:**

This study provides support for the concurrent validity of self-reported upper and lower body muscular fitness assessments (i.e., the push-up and sit-to-stand tests) in mHealth. While these tests may be a feasible option for large scale physical activity interventions, more research is needed to determine the generalisability of these results.

## Background

Given the global uptake of smartphone ownership and their potential for wide reach (1), commercial and research smartphone-based applications (‘apps’) promoting physical activity are being increasingly used [1–4]. App-based interventions (i.e., mHealth interventions) have the potential to influence physical activity behaviour by disseminating information, fostering social support, and applying behaviour change techniques (e.g., self-monitoring, goal setting, action planning) [5–7]. The majority of these mHealth interventions have promoted aerobic physical activity, but few have incorporated resistance training [2–8]. This is surprising given participation in resistance training is a specific component of physical activity guidelines internationally and can provide additional health benefits distinct from those gained through aerobic exercise [9]. Additionally, the prevalence of sufficient resistance training (i.e., 2 or more days/week) among adults is much lower (10–30%) than for aerobic activity (~50%) [9, 10].

mHealth interventions have incorporated self-assessment of physical activity, both as a dedicated behaviour change technique and as a means of determining intervention efficacy [11]. Assuming values obtained through fitness self-assessment are valid, including such a function within mHealth interventions might have the added benefit of enabling feasible and cost-effective evaluation of intervention efficacy/effectiveness. This would be particularly beneficial for dissemination research wherein the resource requirements for researcher-assessed fitness measures are substantial and the costs potentially prohibitive. Despite this promise, there is limited evidence of the validity of muscular fitness self-assessments within mHealth interventions. Such evidence is necessary to inform mHealth interventions with a focus on resistance training, which are new but increasing within the public health literature. For example, ‘ecofit’ is a community-based intervention that integrates resistance- and aerobic-based physical activity through smartphone technology, social support and the use of outdoor exercise equipment [12]. While the *ecofit* intervention has been found efficacious [13, 14], its effectiveness is yet to be determined [12]. Based on the results from the effectiveness trial, the *ecofit* intervention is anticipated to be disseminated among a much larger proportion of participants, and thus will require more feasible data collection methods (i.e., less reliant on face-to-face assessments).

The purpose of this study was to evaluate whether self-assessed muscular fitness can be a valid substitute for researcher-assessed muscular fitness to support the scale-up of the *ecofit* intervention. More specifically, the aim of this study was to assess the concurrent validity of an app-based muscular fitness self-assessment (i.e., measuring upper and lower body strength). We hypothesised moderate to strong correlations between the researcher- and self-assessed push-up and 60-second sit-to-stand tests.

## Materials and methods

### Study design

This correlational study examined baseline data from the *ecofit* randomised controlled trial (RCT). The RCT protocol is published elsewhere [12]. Ethical approval was obtained from the Human Research Ethics Committee of the University of Newcastle, Australia. The *ecofit* trial is registered with the Australian and New Zealand Clinical Trial Registry (ANZCTR): ACTRN12619000868189.

### Participants

Adults (aged 18–80; *N* = 245) not meeting the current aerobic and/or resistance-based physical activity guidelines (i.e., 150–300 minutes of moderate-to-vigorous physical activity and minimum two sessions of muscle strengthening exercises per week) [9] based on self-reported physical activity were recruited on a rolling basis, with interruptions due to the COVID-19 pandemic between October 2019 and March 2021. Participants randomised to receive the *ecofit* intervention (*n* = 122) completed face-to-face, researcher-administered fitness tests as part of baseline assessments and were provided with the purpose-built *ecofit* smartphone application directly thereafter, which included a purpose-designed fitness self-assessment function. Hereafter, the self-assessments completed independently by participants via the app are referred to as ‘self-assessed’, whereas assessments conducted by the researchers are referred to as ‘researcher-assessed’.

### Procedure

The researcher-assessed fitness tests were undertaken at a university laboratory, where participants’ upper and lower body muscular fitness were assessed by a trained researcher using validated measures. Randomisation took place after participants had attended their baseline assessment (i.e., intervention group or wait-list control group). The intervention group received access to the app once they had attended a compulsory 90-minute introductory session, which provided them with instructions on how to operate the app. (See Table 1 for the time-lag between the baseline assessment and the introductory session.) During the introductory session, participants were shown how to perform each exercise and conduct the self-assessments (i.e., record the assessments in the app) before they attempted their first *ecofit* workout (i.e., an app-facilitated session using freely available outdoor exercise equipment). For a detailed description of the face-to-face assessment procedure, see the original trial protocol [12].

**Table 1:** Participant characteristics, baseline results and study lag for the sit-to-stand test.

	Completed the sit-to-stand test within 14-days (n = 54)	Did not attempt the sit-to-stand test (n = 54)	p-value
Male/female, n (%)	18 (33.3%)/ 36 (66.7%)	15 (27.8%)/ 39 (72.2%)	.531
BMI (mean, SD)	28.9 (6.1)	29.1 (6.2)	.875
Age (mean, SD)	55.6 (14.2)	53.2 (14.0)	.373
Research assessed Sit-to-stand test (mean, SD)	24.0 (6.0)	26.7 (9.7)	.143
Lag between baseline assessment and receiving access to the app, mean days (SD)	24.4 (10.0)	-	-
Highest education completed, n (%):			.831
• Graduate school	9 (16.7%)	10 (18.9%)	
• Some Graduate school	1 (1.9%)	2 (3.8%)	
• College/University	28 (51.9%)	26 (49.1%)	
• Some College/University	10 (18.5%)	7 (13.2%)	
• High school	4 (7.4%)	7 (13.2%)	
• Primary school	2 (3.7%)	1 (1.9%)	
Income per household, n (%):			.745
• <$20,000	3 (5.6%)	3 (5.8%)	
• $20,000–39,999	6 (11.1%)	5 (9.6%)	
• $40,000–59,999	9 (16.7%)	6 (11.5%)	
• $60,000–79,999	5 (9.3%)	8 (15.4%)	
• $80,000–99,999	5 (9.3%)	2 (3.8%)	
• >$100,000	26 (48.1%)	28 (53.8%)	
Employment status, n (%):			.141
• Full-time	18 (33.3%)	18 (34.0%)	
• Part-time	13 (24.1%)	6 (11.3%)	
• Casual	3 (5.6%)	3 (5.7%)	
• Volunteer	3 (5.6%)	0 (0%)	
• Retired	14 (25.9%)	15 (28.3%)	
• Other	3 (5.6%)	9 (12.9%)	
• Unemployed	0 (0%)	2 (3.8%)	

### Researcher-assessed fitness assessment

Upper body muscular endurance was measured using the validated 90-degree push-up test [15]. One push-up repetition consisted of lowering the body until the elbows bent 90-degrees and the upper arms were parallel to the floor, while keeping the correct posture (i.e., straight line from the toes to hips and to the shoulders), followed by pushing back up to the starting position. Participants were instructed to perform as many push-ups on their toes (i.e., no option to complete on knees) as they could without breaking form for more than two consecutive or non-consecutive push-ups, in rhythm with a metronome (set at 40-beats/minute) [15]. Lower body muscular endurance was assessed using the validated 60-second sit-to-stand test protocol [16]. The sit-to-stand test involves participants standing up and sitting down on a chair of fixed height as many times as possible within one minute [17]. The number of repetitions completed for each test was recorded by a research assistant.

### Self-assessed fitness assessment

**Fig 1:**
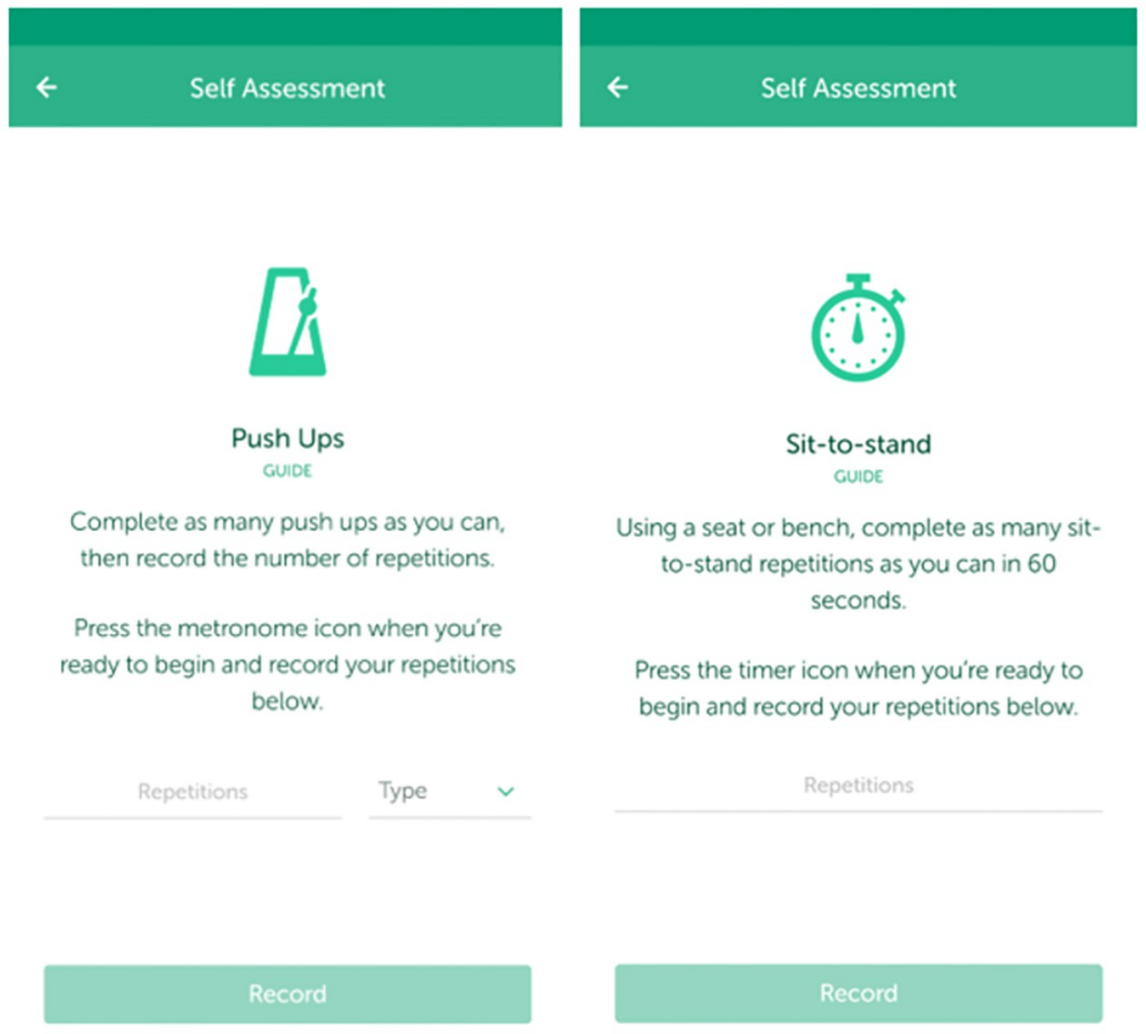
Screen shot of where participants record the push-up and sit-to-stand fitness tests.

**Fig 2:**
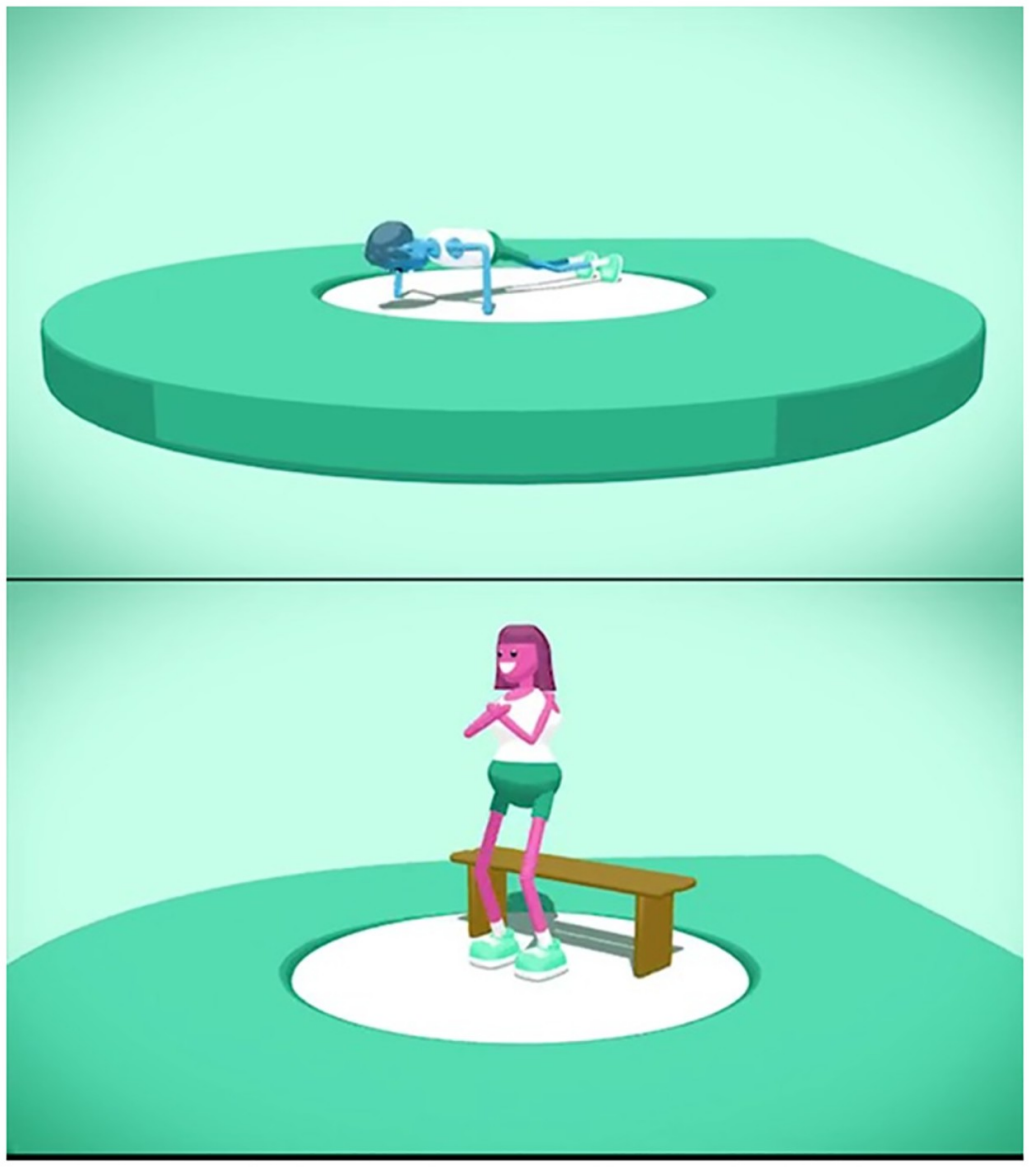
Screen shot of the animated videos demonstrating how to perform the push-ups (left picture) and sit-to-stand (right picture).

The self-assessment aimed to measure participants’ upper and lower body muscular fitness using the same tests as those conducted during the baseline assessment (i.e., push-ups and sit-to-stand). The app-based push-up test included a recording of a metronome at 40-beats/minute, which participants were instructed to use during the test. In contrast to the research-assessed push-up test, the self-assessed assessment allowed participants to complete push-ups on either their knees or toes. For the sit-to-stand test, the app included a built-in 60-second timer and participants were required to count their own repetitions. After each test, participants manually entered the number of repetitions they completed in the app and for the push-up test, entered what option they chose (i.e., toes, knees; see Fig 1). All self-assessment measures included an animated video and written instructions on how to correctly perform the test (see Figs 1 and 2).

### Data analysis

**Fig 3:**
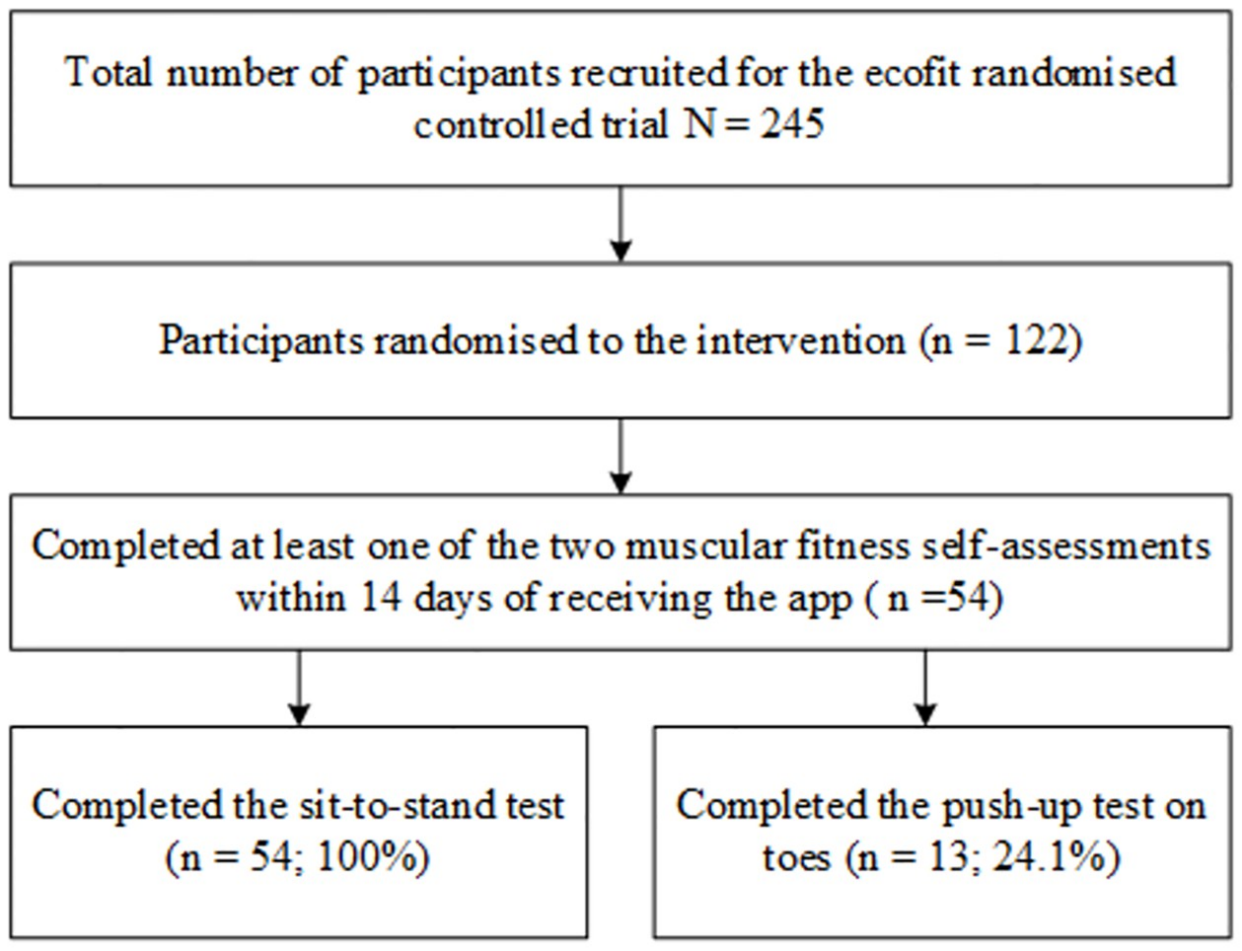
Flow diagram of participants.

All analyses were performed using IBM SPSS Statistics (Version 27). Statistical significance was set at *p*<0.05. Descriptive statistics are reported as frequency or mean ± SD, as appropriate. The sample for the present study were those that completed researcher-assessed tests and baseline and at least one of the two self-assessment muscular fitness tests (i.e., sit-to-stand test, push-up test) within 14-days of receiving access to the *ecofit* app (see Fig 3). For the self-assessed push-ups, only push-ups completed on toes were included in the analysis (as indicated by participants from the app entries). Spearman’s rank correlation coefficient was used to examine the validity between participants’ performance on the research-assessed and self-assessed fitness tests. The strength of the correlation was considered negligible (<0.3), weak (0.31–0.50), moderate (0.5–0.7), strong (0.7–0.9), or very strong 0.91–1.0 [18, 19]. Intraclass correlation coefficients (ICC) were also calculated to determine the reliability between the researcher- and self-assessed fitness tests. Values were considered excellent (>0.9), good (0.75–0.9), moderate (0.5–0.75) or poor (<0.5), based on 95% confidence intervals (CI) of the ICC estimate [20].

Bland-Altman plots were constructed for the sit-to-stand and push-up tests to allow visualisation of the differences between the research- and self-assessed results (*Y*-axis) against the mean of the differences between the research- and self-assessed results (*X*-axis), following the Bland-Altman method [21]. Means and SDs of the differences were also computed. Upper and lower limit of agreement (LoA) were calculated for both the sit-to-stand and push-up tests.

## Results

**Fig 4:**
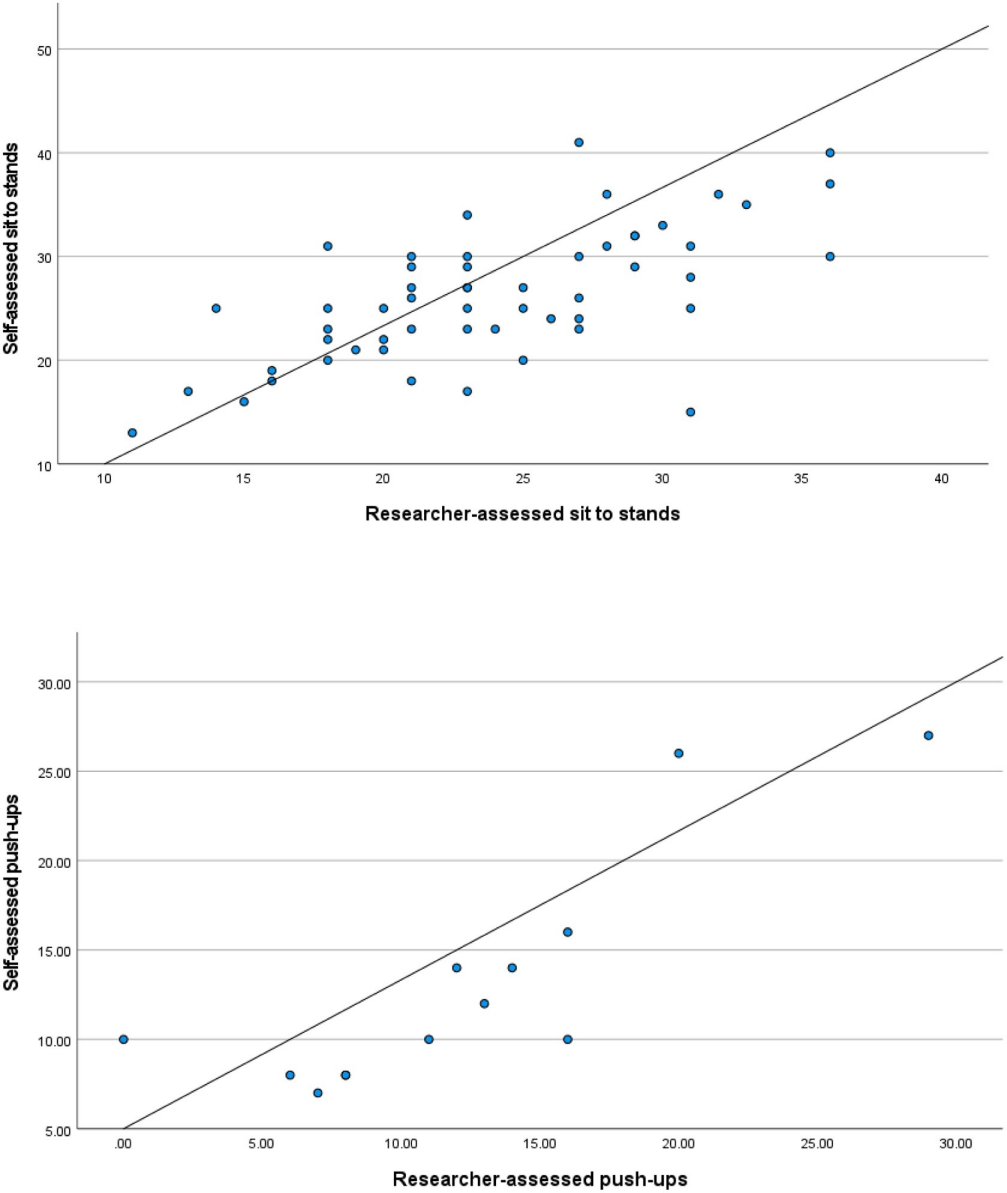
Correlations between the self-assessed and the research assessed sit-to-stand and push-up tests.

Demographic information of participants who either completed or did not complete the self-assessed tests within 14-days can be found in Tables 1 and 2. Of the participants completing the push-up test, (n = 30) were omitted due to completing the self-assessed push-up test on their knees. Spearman’s correlation coefficient was moderate for the sit-to-stand test (0.63, 95% CI 0.40, 0.79, *p* < 0.001) and strong for the push-up test (0.83, 95% CI 0.48, 0.99, *p* < 0.001). The correlations between the self-assessed and research assessed sit-to-stand and push-up test are depicted in Fig 4. The ICCs between the two assessment methods were 0.84 (95% CI = .57 to .95) for the push-up test and 0.65 (95% CI = 0.46 to 0.78) for the sit-to-stand test, and considered ‘good’ and ‘moderate’, respectively.

**Table 2:** Participant characteristics, baseline results and study lag for the push-up test.

	Completed the push-up test on toes within 14-days (n = 13)	Did not attempt the push-up test (n = 60)	*p*-value*	Completed the push-up test on knees within 14-days (n = 30)	*p*-value**
Male/female, n (%)	10 (76.9%)/ 3 (23.1%)	15 (25%) / 45 (75%)	< .001	7 (23.3%) / 23 (76.7%)	< .001
BMI (mean, SD)	27.2 (4.8)	29.3 (6.5)	.271	28.9 (6.4)	.399
Age (mean, SD)	45.6 (12.7)	54.3 (13.9)	.041	56.5 (14.1)	.021
Research assessed push-up test, count (mean, SD)	12.3 (7.2)	3.9 (6.6)	< .001	1.6 (2.6)	< .001
Lag between baseline assessment and receiving access to the app, mean days (SD)	23.5 (7.0)	-	-	-	-
Highest education completed, n (%):			.414		.130
• Graduate school	3 (23.1%)	10 (16.9%)		5 (16.7%)	
• Some Graduate school	0 (0%)	2 (3.4%)		1 (3.3%)	
• College/University	4 (30.8%)	28 (47.5%)		19 (63.3%)	
• Some College/University	5 (38.5%)	9 (15.3%)		2 (6.7%)	
• High school	1 (7.7%)	8 (13.6%)		2 (6.7%)	
• Primary school	0 (0%)	2 (3.4%)		0 (0.0%)	
Income per household, n (%):			.430		.186
• <$20,000	0 (0%)	3 (5.2%)		3 (10.0%)	
• $20,000–39,999	2 (15.4%)	6 (10.3%)		3 (10.0%)	
• $40,000–59,999	1 (7.7%)	8 (13.8%)		5 (16.7%)	
• $60,000–79,999	0 (0%)	8 (13.8%)		4 (13.3%)	
• $80,000–99,999	0 (0%)	3 (5.2%)		3 (10.0%)	
• >$100,000	10 (76.9%)	30 (51.7%)		12 (40.0%)	
Employment status, n (%):			.418		.234
• Full-time	8 (61.5%)	19 (32.2%)		7 (23.3%)	
• Part-time	3 (23.1%)	8 (13.6%)		6 (20.0%)	
• Casual	0 (0%)	3 (5.1%)		3 (10.0%)	
• Volunteer	0 (0%)	1 (1.7%)		2 (6.7%)	
• Retired	2 (15.4%)	17 (28.8)		9 (30.0%)	
• Other	0 (0%)	9 (15.3%)		3 (10.0%)	
• Unemployed	0 (0%)	2 (3.4%)		0 (0.0%)	

*Comparing those who completed the push-ups on their toes within the 14-days and those who did not attempt the test at all.

**Comparing those who completed the push-ups on their toes and those who completed the push-ups on their knees within the 14-days.

**Fig 5:**
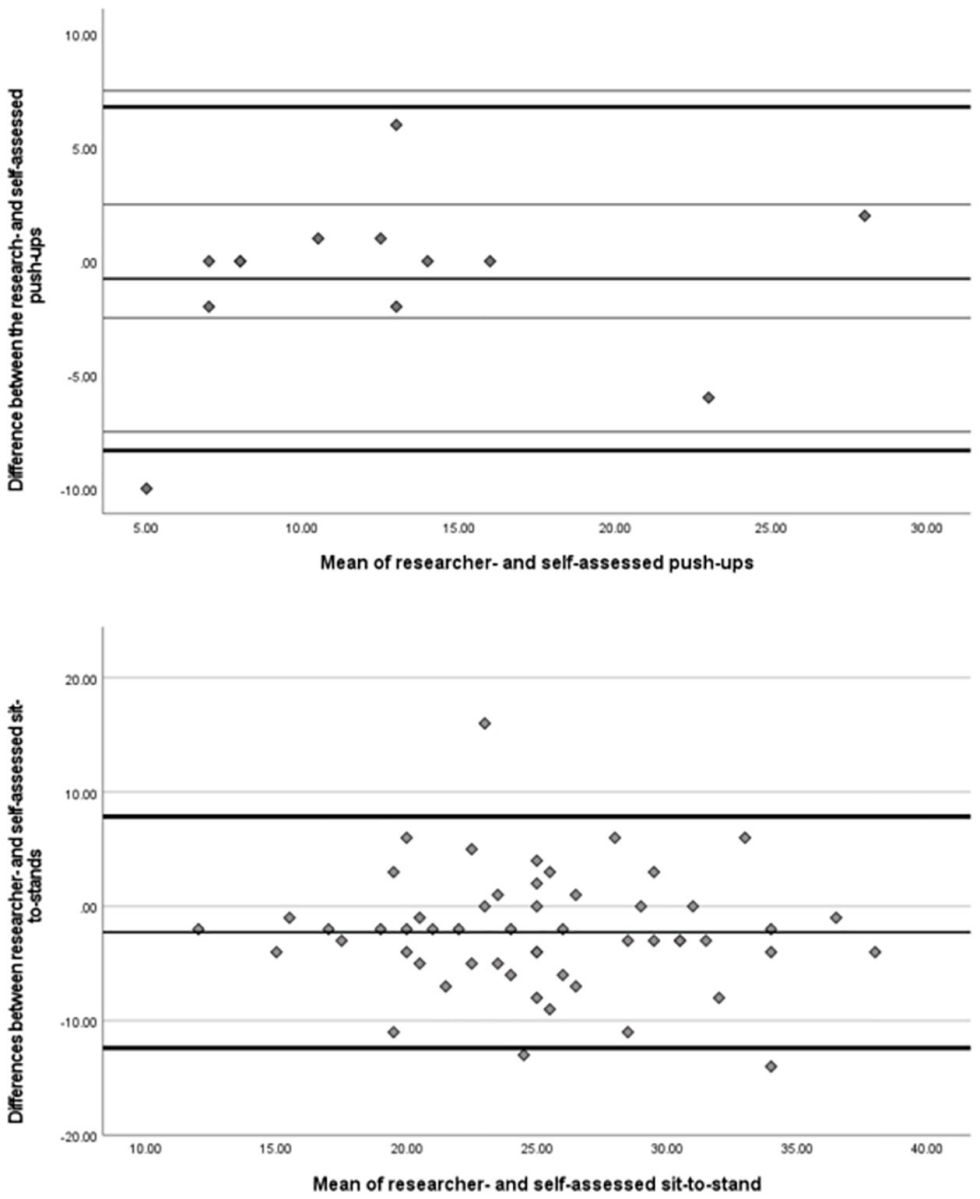
Bland-Altman plots showing differences between researcher-assessed and self-assessed data for the (a) push-up (M = -0.79) and (b) sit-to-stand tests (M = 2.26), with 95% limits of agreement (thick black lines). The means for each test are depicted with a thin black line.

Fig 5 shows the Bland-Altman plots, and illustrates the individual differences between the researcher- and self-assessed fitness for both the sit-to-stand and push-up tests. The mean of the differences between the self- and the researcher-assessed sit-to-stand test was -2.26 (SD = 5.16, 95% LoA 7.85, -12.37), with higher values in the self-assessed tests. Similarly for the push-up test, the mean difference was -0.77 (SD = 3.85, 95% LoA 6.79, -8.32), i.e., higher scores in self-assessed fitness tests. Bland Altman plots showed that most differences were within the limits of agreement but there was a small degree of systematic bias, i.e., over-estimating outcomes when self-reporting. The relationship between the mean differences and the means of the researcher- and self-assessed fitness for the sit-to-stand (r = 0.006, *p* = 0.89) push-ups (r = 0.18, *p* = 0.41) were weak and not significant, indicating that the magnitude of this bias is small and negligible.

## Discussion

The aim of the present study was to assess the concurrent validity of two self-assessed muscular fitness tests (i.e., the push-up and sit-to-stand tests), by comparing self- and researcher-assessed baseline results of the *ecofit* trial [12]. This study found a strong and significant correlation between the researcher- and self-assessed values on the push-up test and a moderate significant correlation between researcher- and self-assessed values on the sit-to-stand test. The Bland-Altman plots showed that mean differences for both fitness tests were close to zero and within the limits of agreement, indicating only a small amount of systemic bias. Further, the relationship between the mean differences and the means of the researcher- and self-assessed fitness for both muscular fitness tests were not significant, indicating that agreement was similar across the range of values observed. As such, we found evidence to support the notion that the sit-to-stand and push-up tests can yield largely valid estimates of upper and lower body muscular fitness when self-administered by participants.

To our knowledge, no previous studies have examined the concurrent validity of the self-administered sit-to-stand and push-up tests using mHealth in the general population. Rees-Punia and colleagues, conducted a study to test the validity between self-reported and video-observed 30-second sit-to-stand test in a population of 134 adults. The authors found no significant difference between self-reported and video-observed number of sit-to-stands, and a strong correlation (ρ = 0.97) between the two assessment methods [22]. Moreover, the validity of self-assessed measures of balance, and functional and exercise capacity has also been established with older people and clinical populations (e.g., Parkinson’s disease [23], multiple sclerosis [24]). Assessments such as the Six-minute Walk Test and 5 U-Turn Test have also demonstrated to be valid tools that can be self-administered via mHealth [24, 25].

The correlation between the research- and self-assessed values on the push-up test were considered strong, however, it appears that participants who completed the push-up test on toes had a higher baseline value than those not attempting the test or who completed the push-up test on their knees. It is possible that the self-assessed push-up test on toes is more suitable towards individuals who are able to perform push-ups. Males were more likely to complete the push-ups on their toes than females, and more females chose not to attempt the self-assessed push-up test at all. It may be that for individuals who are unable to perform push-ups on toes, particularly females, this test may not be appropriate. In addition, those who completed the push-ups on toes were younger, compared to those who did not attempt the push-up test or completed the push-ups on knees. Future studies should investigate the inclusion of self- versus research-assessed push-up test on knees or other upper body muscular fitness tests that can be self-administered, which requires less muscular fitness than push-ups on toes.

The main strength of this study is that it adds to the literature in terms of self-assessed muscular fitness testing. These findings are promising given the potential of using these assessments as a proxy for researcher-assessed fitness testing, and thus enable cost-effective evaluation in the planned scaled-up version of *ecofit* [12]. Being able to continue to evaluate intervention effectiveness will be useful in assessing potential ‘voltage drop’ of the *ecofit* program, i.e., where interventions are projected to produce lower effects as they move along the research translational continuum from efficacy towards community-wide dissemination [26]. Dissemination research typically aims to measure the process of change and assess the reach of the program across the included settings and populations, often assume continued efficacy/effectiveness based on past trials [27]. Adaptions that assist the expansion of an evidence-based program are often needed and frequently undertaken when scaling-up interventions [28]. Being able to administer valid self-assessed fitness tests will be useful in resistance training interventions targeting rural and remote settings, particularly when delivered at scale, where researcher-assessed fitness testing may not be geographically feasible. This would allow participants to complete the assessments at times and locations convenient to them.

Limitations of the study include the relatively low uptake of the self-assessment with less than 50% of the total number of participants completing at least one of the two muscular fitness assessment within 14-days of receiving the app. Including more reminders in the smartphone application to complete the self-assessment, may have been beneficial to increase fitness test self-completion. Another potential limitation of the study was that the chair height in the sit-to-stand test was not standardized between the researcher and participant-conducted protocols, which may have limited the magnitude of the correlation between the two assessment methods. Indeed, the correlation between the two methods may have been even greater if this was done. A further limitation of this study was that participants had the option to complete the push-up test on their knees in the self-assessment and those who completed the app-based test on their knees were omitted from this study to maximise comparability between the app and researcher tests. Future studies are encouraged to ensure that both the researcher- and self-assessed tests are carried out in the same manner and to evaluate the validity of tests conducted on knees and toes. This study is based on a secondary analysis, which was initially powered for the ecofit randomised controlled trial. We acknowledge the sample size of the current study subset is smaller than that of the main study. Because of the small homogeneous sample, it is also unclear how generalisable these results are, as such, more studies are needed in diverse subpopulations and settings. Given the low participation in completing push-ups on toes, future studies should include the option to complete the push-ups on knees. Future studies may also explore other muscular fitness test that do not require additional equipment.

## Conclusion

This study provides support for the concurrent validity of self-reported muscular strength assessments in mHealth. These self-assessed tests may be a feasible option for large scale, population-based studies. The sit-to-stand test is appropriate to be used in app protocols in assessing lower body strength with the general population. However, more work is required to determine the generalisability of the push-up test.
